# Early decrease in postoperative serum albumin predicts severe complications in patients with colorectal cancer after curative laparoscopic surgery

**DOI:** 10.1186/s12957-018-1493-4

**Published:** 2018-09-25

**Authors:** Yong Wang, Honggang Wang, Jianguo Jiang, Xiaofei Cao, Qinghong Liu

**Affiliations:** Department of General Surgery, Taizhou People’s Hospital, Taizhou Clinical Medical College of Nanjing Medical University, Medical School of Nantong University, No.366 Taihu Road, Taizhou, 225300 Jiangsu China

**Keywords:** Colorectal cancer, Postoperative complications, Predictor, Albumin

## Abstract

**Background:**

Postoperative severe complications are always associated with prolonged hospital stays, increased economic burdens, and poor prognoses in patients with colorectal cancer (CRC). This present study aimed to investigate potential risk factors including serum albumin (Alb) for severe complications in CRC patients.

**Methods:**

Eligible patients with primary CRC undergoing elective laparoscopic colectomy from July 2015 to July 2017 were included. Postoperative severe complications were defined as grade III and IV according to the Clavien–Dindo classification. ∆Alb was defined as (preoperative Alb − nadir Alb within POD2)/preoperative Alb × 100%. The baseline characteristics, intraoperative data, and laboratory data were obtained from the database for the analysis. Univariate and multivariate logistic regression analyses were utilized for the assessment of the association between risk factors and postoperative severe complications. The predictive value of ∆Alb for postoperative severe complications was evaluated by receiver operating characteristic (ROC) curve analysis.

**Results:**

A total of 193 patients were finally included in the analysis data set, of which 38 (19.7%) patients had postoperative severe complications. In the final multivariate logistic regression analysis, ∆Alb was the only independent factor associated with postoperative severe complications (OR 1.66, 95%CI 1.18–2.33, *p* = 0.003). The area under the curve (AUC) of ∆Alb was 0.916, with the sensitivity and specificity of 0.842 and 0.858 (*p* < 0.001).

**Conclusions:**

The ∆Alb was an independent risk factor for severe complications in CRC patients after curative laparoscopic surgery.

## Background

Colorectal cancer (CRC) has been widely accepted as the third most common malignant neoplasm worldwide with an increasing incidence in recent years [1]. Surgical resection remains the cornerstone curative treatment for CRC, and laparoscopic colorectal surgery is developing constantly in the recent decades. Despite the great improvements in surgical procedures, perioperative managements, and multidisciplinary therapies, postoperative severe complications persist to some extent [2]. As illustrated by the previous data, the incidence of postoperative severe complication can reach as high as approximately 25% [3]. The severe complications are always associated with prolonged hospital stays, increased economic burdens, and poor prognoses [4]. Therefore, to investigate potential factors for postoperative severe complications can help to stratify complication risks and improve clinical decision-making.

After surgery, circulating acute phase proteins, such as interleukin-6 (IL-6) and C-reactive protein (CRP), usually increase because of the surgical stress and proinflammatory cytokines [5, 6]. Previous data has widely considered these two proteins as potential predictors for postoperative complications after elective colorectal surgery [7]. Albumin (Alb), as a negative acute phase protein and nutritional marker, decreases immediately after operation in response to surgical stress. Previous literature has suggested the prognostic role of preoperative hypoalbuminemia in patients undergoing colorectal surgery [8]. However, few studies have focused on the effect of the change of serum Alb on postoperative complications. This present study aimed to investigate potential risk factors including proinflammatory cytokines and nutritional markers for severe complications in CRC patients.

## Methods

### Patients

This retrospective study protocol was approved by the Medical Institutional Ethics Committee of Jiangsu province. Eligible patients with primary CRC undergoing elective laparoscopic colectomy at the Department of General surgery, Taizhou People’s Hospital from July 2015 to July 2017 were included. The inclusion criteria were as follows: (1) adult patients aged over 18, (2) first pathologically diagnosed with primary CRC supported by operative and pathological results, and (3) patients who underwent a curative laparoscopic resection of primary tumors for the first time. The exclusion criteria were as follows: (1) with tumor metastasis found either pre-operatively or intra-operatively, (2) with emergency operation due to complications (bowel obstruction, perforation, etc.), (3) with neo-adjuvant treatment, (4) accompanied by other malignancies; (5) and with laparotomy or laparoscopic conversion to laparotomy.

### Study design

The surgical procedures, including the extent of both colectomy and lymph node dissection, were conducted according to the Colorectal Cancer Treatment Guidelines [9]. All the enrolled patients received the same perioperative managements. The diet was not resumed until the patients passed flatus. No patients died within postoperative day (POD) 30 in this present study.

The baseline characteristics (age, gender, etc.), intraoperative data (duration of operation, intraoperative blood transfusion, etc.), and laboratory data (CRP, Alb, etc.) were obtained from the database for the analysis. The pathological classifications were evaluated following the guidance of the 7th edition of American Joint Committee on Cancer (AJCC) TNM Classification.

### Definitions and outcomes

The primary outcome was the occurrence of postoperative complications within postoperative 30 days [10]. Postoperative severe complications were defined as grade III and IV according to the Clavien–Dindo classification [11]. Enrolled patients were initially grouped according to the presence of severe complications.

As reported by previous studies [12], the relative change of the serum Alb (∆Alb) was defined as (preoperative Alb − nadir Alb within POD2)/preoperative Alb × 100%. The relative changes of the hemoglobin (Hb) and hematocrit (Hct) were with the same definitions. The median ∆Alb level was accepted as the cutoff value for the discrimination of high versus low value [7].

### Statistical analysis

Data were analyzed by the SPSS 23.0 (SPSS, Inc., IA, USA) and GraphPad Prism 5.0 (GraphPad Inc., CA, USA), and a *p* < 0.05 was considered statistically significant. Before the study, we performed a sample size estimation of 150 patients. According to our clinical experience and previously published reports [7], the estimated incidence of postoperative severe complications was used as a basis for the minimum sample size estimation. Categorical data are presented as a number with percentage, whereas quantitative data is presented as median (range) or mean ± standard error (SE) respectively. Mann–Whitney *U* test or Student *t* test was used for continuous variables analysis, whereas chi-square test or Fisher’s exact test was used for categorical variables analysis as appropriate. Only those potential risk factors (*p* < 0.05) on univariate analysis were enclosed into the final multivariate logistic regression analysis. Binary multivariate stepwise logistic regression model was used in this study. The continuous data used in the logistic model was divided into two groups (high vs low, using the median value as the cutoff value). The predictive value of ∆Alb for postoperative severe complications was evaluated by receiver operating characteristic (ROC) curve analysis.

## Results

### Patient characteristics

**Fig. 1:**
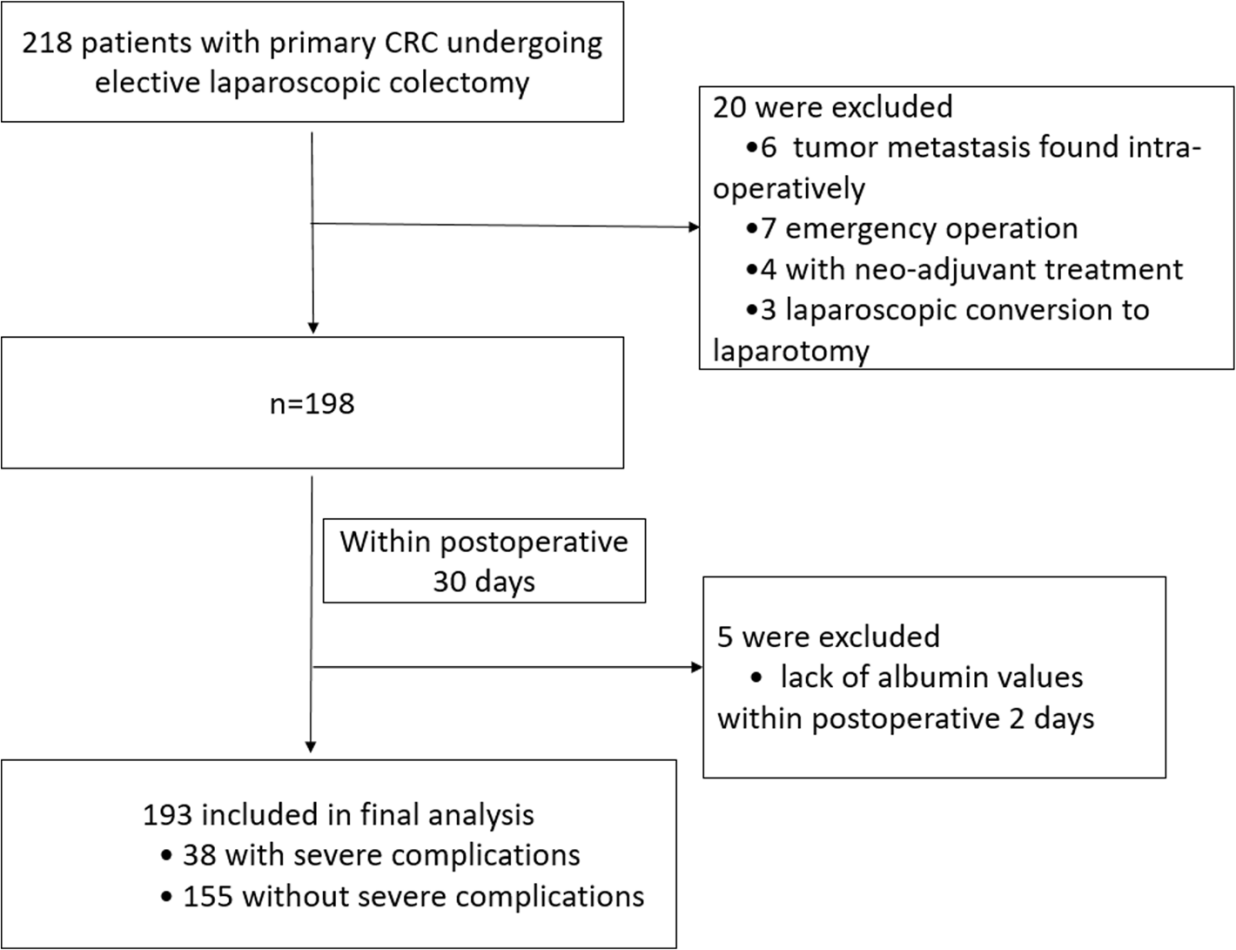
Flow chart of the cases analyzed

Of 218 consecutive patients, 25 patients were excluded according to the exclusion criteria (6 tumor metastases found intra-operatively, 7 emergency operations, 4 with neo-adjuvant treatment, 3 laparoscopic conversions to laparotomy, and 5 lack of albumin values within postoperative 2 days), which is shown in Fig. 1. A total of 193 patients were finally included in the multivariate analysis dataset, of which 60.1% (116/193) were male patients, as shown in Table 1. Eventually, 38 patients had postoperative severe complications according to the Clavien–Dindo grade, with an incidence of 19.7%. Postoperative severe complications included 6 severe bleedings, 9 anastomotic leakages, 18 severe infections, 4 bowel obstructions, and 1 severe cardiopulmonary failure. The mean age and BMI of the total cohort were 53.4 years and 20.8 kg/m^2^ respectively. Patients with severe complications had an older age than those without severe complications (52.5 ± 11.7 vs 57.2 ± 12.1, *p* = 0.029). Patients with the comorbidity of hypertension were at an increased risk of postoperative severe complications (*p* = 0.029). The history of previous abdominal surgery was also significantly associated with increased severe complications (*p* = 0.030). In addition, patients with severe complications had a longer duration of operation (*p* = 0.043) and more estimated intraoperative blood loss (*p* = 0.032). Those patients with perioperative blood transfusion were also frequent in the patients with severe complications (*p* = 0.026). No significant differences were found in gender, BMI, smoking habits, ASA class, tumor location, AJCC stage, intraoperative fluid utilization, number of lymph nodes resection, and time to first flatus between the patients with or without severe complications (all *p* > 0.05).

**Table 1:** Clinicopathological characteristics of CRC patients with severe complications or not

Parameters	Postoperative severe complications		*p* value
	No (*n* = 155)	Yes (*n* = 38)	
Age (year)	52.5 ± 11.7	57.2 ± 12.1	0.029*
Gender, *n* (%)			
Male	92 (59.4)	24 (63.2)	
Female	63 (40.6)	14 (36.8)	0.67
BMI (kg/m^2^)	20.9 ± 1.3	20.6 ± 1.5	0.218
Comorbidities, *n* (%)			
Hypertension	28 (18.1)	13 (34.2)	0.029*
Diabetes mellitus	18 (11.6)	6 (15.8)	0.48
Smoking status, *n* (%)			
Current smoker	17 (11.0)	7 (18.4)	
History of smoking	14 (9.0)	5 (13.2)	
Never	124(80.0)	26(68.4)	0.30
ASA class, *n* (%)			
II	121 (78.1)	28 (73.7)	
III	34 (21.9)	10 (26.3)	0.56
Previous abdominal surgery, *n* (%)	38 (24.5)	16 (42.1)	0.030*
Tumor location, *n* (%)			
Colon	90 (58.1)	20 (52.6)	
Rectum	65 (41.9)	18 (47.4)	0.54
AJCC stage, *n* (%)			
I–II	88 (56.8)	18 (47.4)	
III	67 (43.2)	20 (52.6)	0.30
Duration of operation (min)	203.4 ± 30.1	215.4 ± 41.2	0.043*
Estimated blood loss (mL)	180 (60–710)	240 (80–850)	0.032*
Intraoperative fluid utilization (mL)	1800 (1300–3100)	1900 (1400–2900)	0.28
Number of lymph nodes resection	10.7 ± 5.2	10.9 ± 5.8	0.836
Perioperative blood transfusion, *n* (%)	34 (21.9)	15 (39.5)	0.026*
Time to first flatus (d)	3.0 ± 0.6	3.1 ± 0.9	0.410

*CRC* colorectal cancer, *BMI* body mass index, *ASA* American Society of Anesthesiologists, *AJCC* American Joint Committee on Cancer. *p* values were calculated by chi-square test, Fisher’s exact test, Mann–Whitney *U*, or *t* test. *p value < 0.05

### Laboratory tests

As summarized in Table 2, the patients with severe complications had a significantly higher ∆Alb value than those without severe complications (13.3 ± 2.9 vs 19.0 ± 3.5, *p* < 0.001). In addition, higher ∆Hb (*p* = 0.034) and peak CRP level within POD3 (*p* = 0.005) were also significantly associated with postoperative severe complications.

**Table 2:** Laboratory tests in CRC patients with severe complications or not

Laboratory tests	Postoperative severe complications		*p* value
	No (*n* = 155)	Yes (*n* = 38)	
Preoperative Hb	117.5 ± 7.5	116.4 ± 8.4	0.43
Preoperative Alb	39.2 ± 4.6	37.8 ± 5.1	0.10
Preoperative Hct	0.42 ± 0.07	0.43 ± 0.05	0.41
Preoperative CRP	10.8 ± 3.5	11.1 ± 2.7	0.62
∆Hb (%)	14.1 ± 4.2	15.7 ± 3.9	0.034*
∆Alb (%)	13.3 ± 2.9	19.0 ± 3.5	< 0.001*
∆Hct (%)	− 10.2 ± 12.4	− 11.4 ± 13.6	0.60
Peak CRP within POD3	89.4 ± 6.8	104.4 ± 9.1	0.005*

*CRC* colorectal cancer, *Hb* hemoglobin, *Alb* albumin, *Hct* hematocrit, *CRP* C-reactive protein, *POD* postoperative day. *p* values were calculated by Mann–Whitney *U* or *t* test. *p value < 0.05

### Risk factors associated with postoperative severe complications

Subsequently, univariate and multivariate analyses were performed to investigate potential risk factors for postoperative severe complications. These nine potential risk factors mentioned above (Tables 1 and 2) were enclosed into the univariate analysis. Of the nine factors, five (duration of operation, perioperative blood transfusion, ∆Hb, ∆Alb, and peak CRP within POD3) were significantly associated with postoperative severe complications. In the final multivariate logistic regression analysis, ∆Alb was the only independent factor associated with postoperative severe complications (OR 1.66, 95%CI 1.18–2.33, *p* = 0.003, see Table 3).

**Table 3:** Univariate and multivariate logistic regression analyses of perioperative factors on postoperative severe complications

Variables	Univariate		Multivariate	
	OR (95% CI)	*p* value	OR (95% CI)	*p* value
Age	1.02 (0.98–1.05)	0.75		
Hypertension	1.22 (0.76–1.94)	0.43		
Previous abdominal surgery	1.40 (0.93–2.04)	0.12		
Duration of operation	1.38 (1.03–1.94)	0.043*	0.93 (0.57–1.49)	0.72
Estimated blood loss	0.82 (0.58–1.14)	0.23		
Perioperative blood transfusion	2.46 (1.47–4.02)	0.012*	1.33 (0.62–2.77)	0.43
∆Hb	2.34 (1.29–4.61)	0.009*	1.66 (0.68–3.89)	0.23
∆Alb	1.68 (1.22–2.32)	0.002*	1.66 (1.18–2.33)	0.003*
Peak CRP within POD3	0.70 (0.48–0.99)	0.032*	1.12 (0.65–1.94)	0.65

*Hb* hemoglobin, *Alb* albumin, *CRP* C-reactive protein, *POD* postoperative day, *OR* odds ratio, CI confidence interval. *p value < 0.05

### Predictive value of ∆Alb for postoperative severe complications

**Fig. 2:**
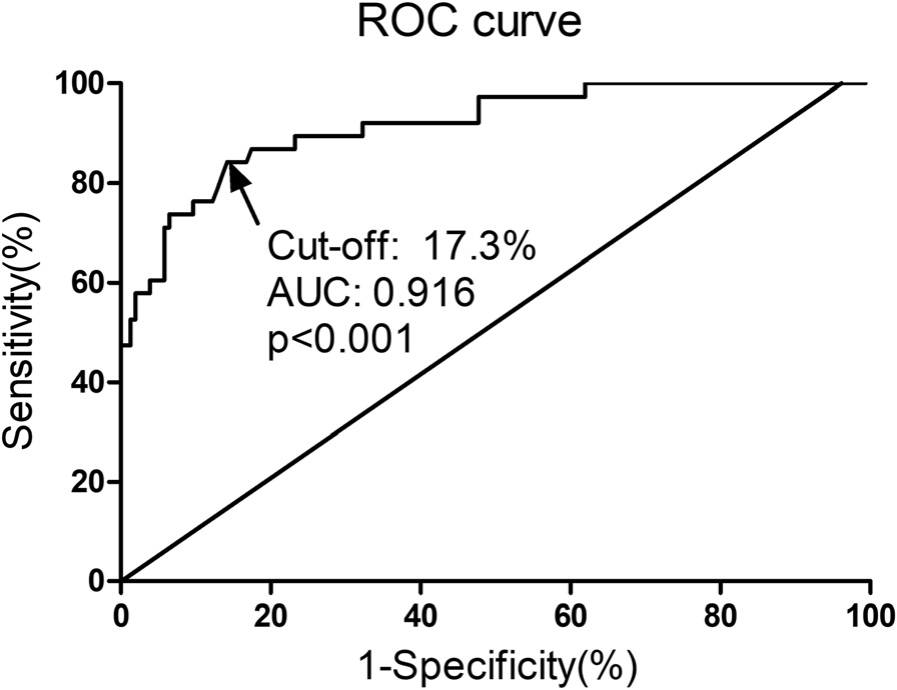
The predictive value of ∆Alb for postoperative severe complications by ROC analysis. AUC 0.916, cutoff value 17.3%, sensitivity 0.842, specificity 0.858, *p* < 0.001. Alb albumin, ROC receiver operating characteristics, AUC area under the curve

The receiver operator characteristic (ROC) curve analysis was applied to establish the predictive power of ∆Alb for postoperative severe complications. As illustrated in Fig. 2, the area under the curve (AUC) of ∆Alb was 0.916, with the cutoff value of 17.3%. The sensitivity and specificity were 0.842 and 0.858, respectively (*p* < 0.001).

## Discussion

In this current study, we focused on the potential association between serum Alb, an acute phase protein, and postoperative severe complications within POD 30. Our results revealed that a greater change in the serum Alb within POD 2 was an independent risk factor associated with postoperative severe complications. Postoperative severe complications were with a rate of 19.7% in this present study, which was relatively similar to previous reports [13].

As reported by previous data, preoperative or early postoperative hypoalbuminemia is accepted as a risk factor for postoperative complications after a gastrointestinal operation, especially surgical site infections [8, 14]. However, whether the relative change in perioperative serum Alb closely correlates with postoperative complications remains unclear. As widely proved, decreased Alb expression after the surgery is always observed due to the systemic inflammatory response syndrome [15]. As illustrated by a recent pilot study, postoperative albumin concentration is significantly decreased and it is suggested as a response biomarker for operation stress [16], which is quite in accordance with our results. In addition, perioperative hemodilution and fluid overload are also important explanations for decreased serum Alb after operation [17]. As summarized by previous studies, the decreased Alb level is ascribed to various factors, including Alb redistribution, perioperative blood loss, catabolism, and hemodilution [17]. A recent study has revealed increased postoperative CRP level as an independent factor associated with Alb reduction [7], which strongly suggests the close association between Alb and inflammatory response. Accumulating evidence has suggested CRP as a predictor for postoperative complications and prognosis after abdominal surgery [18]. Our univariate analysis showed that peak CRP within POD3 was significantly associated with postoperative severe complications; however, the final multivariate results did not suggest its predictive role.

Previous reports have revealed that malnutrition and inflammatory response strongly correlate with severe postoperative complications [19]. As for those patients with malignancy, the development of inflammatory response is closely associated with decreased Alb and total lymphocyte count [20, 21]. Serum Alb is reported to act various roles, including cell growth stabilization, DNA replication, sex hormone homeostasis maintaining, and systemic inflammation modulation[22].

A recent study in patients with cancer has shown that hypoalbuminemia reflects the condition of malnutrition and immunosuppression, and it is at an increased risk of disease severity, tumor progression, and poor prognosis [23]. Furthermore, serum Alb level has also been widely used in various prognostic indexes, including a prognostic nutritional index (PNI) [24], a systematic inflammation index (IPI) [25], and Naples prognostic score (NPS) [26]. Serum Alb plays important roles in colloid osmotic pressure maintenance, free radical scavenging, and capillary membrane permeability alteration [27]. The important physiologic functions of serum Alb may be potential explanations for the predictive role of ∆Alb for postoperative severe complications.

This study has some certain limitations. First, our data set came from a retrospective and single-center design, and the sample size was relatively small. Second, this study did not take the impacts of liver function and body fluid volume on serum albumin concentrations into consideration. Nevertheless, to our knowledge, this is the first study that highlighted the significance of ∆Alb for postoperative severe complications. Of course, additional larger-scale prospective studies and basic researches are needed to confirm our results.

## Conclusions

In conclusion, our results revealed that the ∆Alb was an independent risk factor for severe complications in CRC patients after curative laparoscopic surgery. The surgeon and anesthetist could differentiate the patients according to the reduction trend of serum Alb and treat them accordingly. Predicting the risk of postoperative severe complications with serum Alb detections helps the surgeon in the outcome evaluation. The evaluation of the nutritional status prior to surgery is of great importance and if possible, correcting the deficit is recommended by our results.

## Abbreviations

AJCC: American Joint Committee on Cancer
Alb: Albumin
ASA: American Society of Anesthesiologists
BMI: Body mass index
CI: Confidence interval
CRC: Colorectal cancer
CRP: C-reactive protein
Hb: Hemoglobin
Hct: Hematocrit
OR: Odds ratio
POD: Postoperative day
ROC: Receiver operating characteristic
SE: Standard error
